# Development and psychometric validation of the Problem-Posing Ability Scale for secondary school students

**DOI:** 10.3389/fpsyg.2026.1792188

**Published:** 2026-03-26

**Authors:** Linyu Du, Tingzhao Wang, Mengfei Han, Hongchang Teng, Zhiyue Zhang, Yiwei Yang

**Affiliations:** 1 Faculty of Education, Shaanxi Normal University, Xi’an, China; 2 Department of Education, Ludong University, Yantai, China

**Keywords:** creative thinking, problem-posing ability, psychometric validation, scale development, secondary school students

## Abstract

**Objective:**

This study aimed to develop and validate a Problem-Posing Ability Scale (PPAS) for secondary school students.

**Methods:**

Grounded in the triarchic theory of creative thinking and cognitive process models, initial scale items were generated through literature analysis, semi-structured interviews, and expert review. Using convenience sampling, data were collected in two stages: Item analysis and exploratory factor analysis (EFA) were conducted with a sample of 961 secondary school students. Confirmatory factor analysis (CFA) and criterion-related validity assessment were performed with an independent sample of 577 participants. An additional subsample of 96 students from Sample 2 completed the scale after a 4-week interval to assess test–retest reliability. The Williams Creative Tendency Scale served as the criterion measure.

**Results:**

The final 24-item PPAS comprises four dimensions: Questioning Courage and Initiative, Ability to Articulate and Organize Problems, Depth and Criticality of Thinking, and Problem Awareness and Sensitivity. These factors accounted for 62.5% of the total variance. CFA indicated excellent model fit (χ^2^/df = 1.252, CFI = 0.992, TLI = 0.991, RMSEA = 0.021). The scale demonstrated strong internal consistency (total Cronbach’s α = 0.94; subscale α = 0.875–0.895), high test-retest reliability (*r* = 0.955), and strong criterion-related validity with the Williams Creative Tendency Scale (*r* = 0.903).

**Conclusion:**

The PPAS is a psychometrically sound instrument for assessing problem-posing ability among Secondary school students, with potential applications in educational research and instructional practice.

## 1 Introduction

As Albert Einstein famously noted, “It is often more important to ask a question than to solve a problem.” Question-posing constitutes a foundational process for knowledge construction, self-directed learning, and innovation (Lukas, 2022). Problem-posing ability (PPA) refers to an individual’s capacity to identify cognitive or practical gaps within a given context and to formulate clear, novel questions through creative thinking (Grundmeier, 2015). From the Socratic “midwifery” to contemporary student-centered pedagogies, questioning remains central to cognitive development (Liu and Liu, 2021).

UNESCO has explicitly emphasized that future education should prioritize cultivating the ability to ask valuable questions, as it serves not only as a crucial indicator of higher-order thinking (Brown and Walter, 2005), but also underpins the capacity to navigate uncertainty and engage in lifelong learning (Cai and Wang, 2022). Empirical studies indicate that PPA significantly predicts academic achievement, problem-solving skills, deep knowledge construction, and lifelong learning capacity (Rosli et al., 2013). Granting students an active role in questioning enhances learning autonomy (Marbach-Ad and Sokolove, 2000), as questioning itself is an effective strategy for improving knowledge creation and sharing (Denny et al., 2008).

In the current era of generative artificial intelligence (GAI), the educational paradigm is shifting from answer-oriented to question-driven approaches (Zhang G. C. et al., 2025). An individual’s ability to pose high-quality questions to intelligent tools has emerged as a key factor distinguishing learning efficacy and innovative potential (Zhang et al., 2019; Lan and Lin, 2011). Given that GAI functions as a super-producer of answers, the value of its outputs is largely determined by the quality of the questions posed. Only precise, profound, and insightful inquiries can stimulate AI to generate groundbreaking solutions. Consequently, thinking patterns and questioning abilities are evolving into core competencies in the AI age, making accurate assessment and cultivation of PPA theoretically and practically significant.

Secondary school students are at a critical stage in the development of formal operational thinking, during which fostering proactive inquiry is essential (Sun, 2016). However, research indicates that the overall level of PPA among Secondary school students remains moderate (Mei, 2019), with enthusiasm and willingness to ask questions declining as they progress to higher grades (Zhu, 2017). Factors such as academic level, logical thinking ability, confidence, courage to ask, and tolerance for uncertainty significantly influence the depth and complexity of questions posed (Zhang and Tang, 2023). Parenting styles also play a role; students raised under democratic styles score higher than those under authoritarian styles, while traits like shyness and anxiety show negative correlations with PPA (Zhang et al., 2014). Moreover, the manner of using AI tools can stimulate active inquiry and questioning awareness. These findings suggest that PPA development is jointly shaped by cognitive foundations, affective attitudes, and environmental factors, progressing from “daring to ask” to “knowing how to ask” and finally to “asking good questions” (Gao et al., 2025). Early identification and multidimensional assessment of this ability can help break the inertia of passive knowledge reception and transform vague doubts into clear, researchable propositions, laying the groundwork for lifelong learning.

Despite a consensus on the importance of PPA, existing assessment tools exhibit significant limitations. Most instruments focus predominantly on cognitive aspects (e.g., problem awareness) while neglecting non-cognitive factors such as questioning courage, and often fail to incorporate dimensions relevant to emerging technological contexts like human-machine interaction. Moreover, current studies are largely confined to specific subjects (e.g., mathematics, science), lacking coverage of interdisciplinary, AI-mediated, and real-life scenarios. Assessment methods typically involve theoretical discussions of PPA, evaluating indicators such as question diversity, flexibility, complexity, and originality (Chen, 2023), or rely on qualitative models of problem-posing literacy (Hong et al., 2021; Zhou, 2024). It is important to note that problem-posing ability and problem-posing literacy represent distinct constructs (Liu et al., 2025).

The Primary Trait Analysis (PTA), frequently cited as a PPA assessment tool, is essentially a generic rubric for open-ended tasks. Its non-specific assessment function often leads to ambiguous indicators and overly generalized application scenarios, making it ill-suited to the interdisciplinary and comprehensive competency development needs of secondary school students. Additionally, while online systems (e.g., QSIA, PeerWise) have attempted to integrate questioning activities (Rafaeli et al., 2024), they typically over-rely on self-report, neglect peer and teacher evaluations, and suffer from oversimplified assessment processes (Hazeyama and Hirai, 2009). The Question Formulation Technique (QFT), though widely discussed, is fundamentally a structured intervention strategy for teaching students how to ask questions, not an assessment tool for quantifying PPA.

These limitations underscore the need for a new scale that combines theoretical innovation with practical applicability. Therefore, this study aims to develop and validate a standardized PPAS for secondary school students, grounded in the triarchic theory of creative thinking and cognitive process models, and integrating both cognitive and non-cognitive elements. From a practical standpoint, the scale will provide educators with a tool to identify students’ specific weaknesses in PPA and design targeted instructional strategies, while also offering students a basis for self-monitoring and improvement. Ultimately, this work responds to the trend of AI-powered education and seeks to facilitate the implementation of a “question-driven” educational paradigm.

## 2 Materials and methods

### 2.1 Participants

A convenience sampling method was employed. After obtaining informed consent from school administrators, homeroom teachers, students, and their guardians, an electronic questionnaire link was distributed. Students completed the survey online under uniform supervision in classroom settings. All participation was voluntary. Invalid responses (e.g., completion time<3 min, patterned answering) were excluded. A total of 1,538 valid questionnaires were collected, yielding an effective response rate of 95.41%. Data collection was conducted via the Sojump (Questionnaire Star) platform to ensure consistency. The study protocol was approved by the Academic Ethics Committee of Shaanxi Normal University.

- *Sample 1* (for item analysis and EFA): This sample comprised 961 valid questionnaires (effective response rate 95.5%). Demographics: 461 male (48.0%), 500 female (52.0%); 482 junior high school students (50.2%), 479 senior high school students (49.8%); 661 urban (68.8%), 300 rural (31.2%); 89 from key schools (9.3%), 872 from regular schools (90.7%).
- *Sample 2* (for CFA and criterion validity): An independent sample of 577 valid questionnaires was used (effective response rate 95.2%). Demographics: 257 male (44.5%), 320 female (55.5%); 296 junior high school students (51.3%), 281 senior high school students (48.7%); 383 urban (66.4%), 194 rural (33.6%); 49 from key schools (8.5%), 528 from regular schools (91.5%).
- *Sample 3* (for test–retest reliability): A subsample of 96 students from Sample 2 was retested after a 4-week interval.

### 2.2 Scale development

#### 2.2.1 Theoretical framework

The PPAS was developed based on an integrative framework that conceptualizes PPA as a comprehensive psychological construct enabling individuals to perceptively identify cognitive gaps and generate novel, clearly articulated problems. The framework synthesizes insights from five key theoretical domains (see Table 1).

**TABLE 1:** Summary of core components in problem-posing ability measurement.

Related study(s)	Primary dimension (theoretical perspective)	Secondary dimension (measurement component)	Conceptual definition (operationalization)
Balka (1974) Silver and Cai (1996)	Three-dimensional theory of creative thinking	Fluency	Number of valid questions posed
		Flexibility	Diversity and variety of questions
		Originality	Novelty and rarity of questions
Silver (1994) Grundmeier (2015)	Problem-posing Process theory	Detecting Confusion	Perceiving cognitive conflicts in a situation
		Clarifying Needs	Identifying gaps in knowledge or information needs
		Articulating Questions	Formulating questions clearly in language
		Deepening Inquiry	Enhancing inquiry through follow-up questioning
Christou et al. (2005)	Four-stage cognitive process model	Comprehension	Understanding given information and identifying key elements
		Transformation	Translating information into question form
		Editing	Reorganizing and editing information to create new questions
		Selection	Choosing appropriate information to construct questions based on goals
Gao (2016)	Three-stage competency development model	Formation of problem awareness	Sensitivity to context and curiosity
		Development of problem ability	Capacity to transform vague confusion into clear questions
		Refinement of problem articulation	Expressing questions accurately and concisely
Xia et al. (2008) Zheng et al. (2007)	Problem quality evaluation system	Quantity of problems	Number of valid questions and speed of posing
		Variety of problems	Diversity and complexity of question types
		Quality of problems	Solvability, reasonableness, and inquiry value of questions
		Novelty of problems	Originality and uniqueness of questions
Zhou and Lyu (2002)	Comprehensive evaluation methodology	Quantitative evaluation	Quantification based on frequency of information processing and question characteristics
		Qualitative evaluation	Multi-source qualitative assessment combining self-, peer, and teacher evaluations
Zhang et al. (2014)	Non-cognitive influencing factors	Questioning courage	Willingness to overcome psychological barriers and actively express questions
		Initiative	Spontaneous tendency to ask questions without external pressure
		Self-confidence	Belief in and affirmation of one’s own questioning ability
Zhang et al. (2019)	Contemporary competency components	Human-AI interaction ability	Ability to pose high-quality questions to intelligent tools
		Metacognitive regulation	Monitoring, evaluating, and adjusting the questioning process
		Learning beliefs	Beliefs and attitudes toward the value of posing questions
Li (2022)	Developmental characteristics	Contextual sensitivity	Sensitivity to and interpretation of different situations
		Mathematization of real-world problems	Ability to translate real-world problems into mathematical questions
		Cognitive level	Cognitive complexity of questions posed
Gao (2024)	Academically related dimensions	Knowledge application	Ability to apply existing knowledge to pose questions
		Thinking quality	Characteristics of thinking demonstrated during questioning
		Academic performance	Relationship between problem-posing ability and academic achievement

1. The triarchic theory of creative thinking (fluency, flexibility, originality) (Balka, 1974; Silver and Cai, 1996).
2. Christou et al.’s (2005) four-stage cognitive process model (comprehension, transformation, editing, selection).
3. Gao’s (2016) three-stage developmental model (problem awareness formation, problem ability development, articulation refinement).
4. The influence of non-cognitive factors (questioning courage, self-confidence) (Zhang et al., 2014).
5. Emerging dimensions in digital learning environments, particularly the capacity to formulate high-quality inquiries for AI systems (Zhang et al., 2019).

#### 2.2.2 Item generation and refinement

**FIGURE 1:**
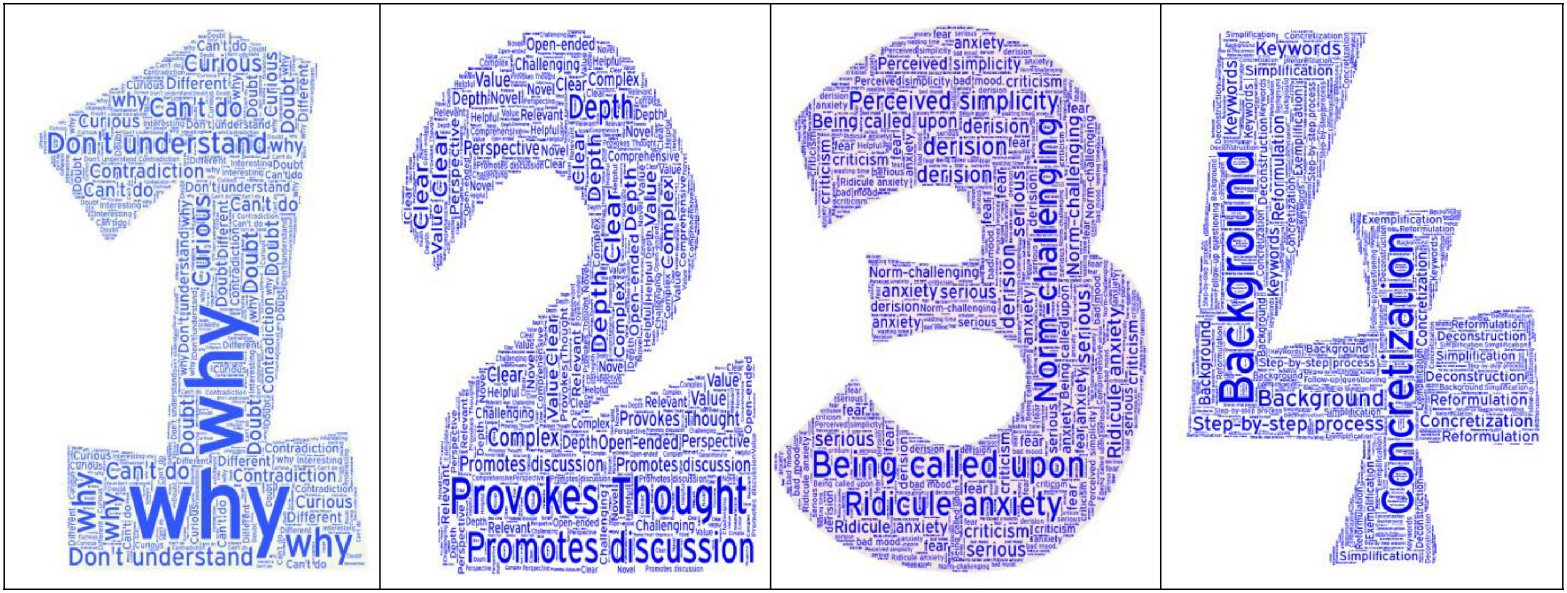
Word frequency analysis map of interview results on secondary school students’ question-posing ability.

An initial pool of items was generated through semi-structured interviews with 20 secondary school students, a comprehensive literature review, and expert consultation. Following a word-frequency analysis of interview transcripts (as shown in Figure 1) and referencing established scales (Silver and Cai, 1996; Ye, 2022), 35 preliminary items were drafted. An expert panel (comprising three education doctoral candidates and five experienced senior high school teachers) rated each item for relevance, clarity, and age-appropriateness. Redundant items were merged, resulting in 24 retained items. Item wording was localized to align with students’ cognitive and cultural context (e.g., “I will conduct in-depth inquiry into cognitive conflicts” revised to “When I encounter a problem I can’t figure out, I keep thinking about it until I understand it”). Contemporary relevance was emphasized by incorporating elements related to human–computer interaction (Ren, 2025).

#### 2.2.3 Preliminary scale

The preliminary PPAS consists of 24 items rated on a 5-point Likert scale (1 = strongly disagree to 5 = strongly agree), with total scores ranging from 24 to 120. Higher scores indicate higher problem-posing ability. To control for response bias, Items 18 and 22 are reverse-scored.

### 2.3 Criterion measure

The Chinese version of the Williams Creativity Assessment Packet (Lin and Wang, 1999) was employed as the criterion measure. It contains 50 items across four dimensions: Risk-taking (11 items), Curiosity (14 items), Imagination (13 items), and Challenge (12 items), rated on a 3-point Likert scale. The scale has demonstrated good psychometric properties in Chinese student populations (total Cronbach’s α = 0.967 in the present study). The conceptual overlap between PPA and creative tendency supports its use for establishing external validity.

### 2.4 Statistical analysis

Data were analyzed using SPSS 27.0 and Amos 24.0. Item analysis included critical ratio (CR) tests and item-total correlations. EFA was conducted using principal component analysis with varimax rotation. CFA employed maximum likelihood estimation. Reliability was assessed via Cronbach’s α coefficient and test-retest correlation. Validity assessments included construct validity (via EFA, CFA, and subscale-total correlations) and criterion-related validity (using Pearson correlation). Continuous variables are presented as the mean (M) ± standard deviation (SD). The significance level was set at α = 0.05 (two-tailed).

## 3 Results

### 3.1 Item analysis

#### 3.1.1 Critical ratio analysis

Based on Sample 1, independent-samples *t*-tests were conducted comparing the high-score group (top 27%) and the low-score group (bottom 27%) for all 24 items. All items showed significantly higher scores in the high-score group (*t* = 14.798–23.480, *p* < 0.001), indicating good discriminative power (see Table 2).

**TABLE 2:** Independent-samples *t*-test results for items between high- and low-scoring groups (*n* = 518).

Item	*t*	df	*p*
A1	16.072	518	< 0.001
A2	21.553	518	< 0.001
A3	19.121	518	< 0.001
A4	20.206	518	< 0.001
A5	15.866	518	< 0.001
A6	20.350	518	< 0.001
A7	17.581	518	< 0.001
A8	20.475	518	< 0.001
A9	14.798	518	< 0.001
A10	20.005	518	< 0.001
A11	20.533	518	< 0.001
A12	18.245	518	< 0.001
A13	16.858	518	< 0.001
A14	23.480	518	< 0.001
A15	20.419	518	< 0.001
A16	21.443	518	< 0.001
A17	17.127	518	< 0.001
A18	21.122	518	< 0.001
A19	20.900	518	< 0.001
A20	16.657	518	< 0.001
A21	14.980	518	< 0.001
A22	20.602	518	< 0.001
A23	21.074	518	< 0.001
A24	21.245	518	< 0.001

#### 3.1.2 Item-total correlation

Correlations between each item and total score ranged from 0.495 to 0.651 (all *p* < 0.001). Cronbach’s α for the total scale was 0.92; deletion of any single item did not increase the α coefficient. Therefore, all 24 items were retained (see Table 3).

**TABLE 3:** Item-total correlations for the Problem-Posing Ability Scale (*n* = 961).

Item	*r*	*p*
A1	0.550	< 0.001
A2	0.627	< 0.001
A3	0.632	< 0.001
A4	0.624	< 0.001
A5	0.518	< 0.001
A6	0.615	< 0.001
A7	0.583	< 0.001
A8	0.580	< 0.001
A9	0.495	< 0.001
A10	0.591	< 0.001
A11	0.640	< 0.001
A12	0.577	< 0.001
A13	0.547	< 0.001
A14	0.651	< 0.001
A15	0.637	< 0.001
A16	0.600	< 0.001
A17	0.556	< 0.001
A18R	0.615	< 0.001
A19	0.628	< 0.001
A20	0.548	< 0.001
A21	0.507	< 0.001
A22R	0.634	< 0.001
A23	0.650	< 0.001
A24	0.627	< 0.001

### 3.2 Exploratory factor analysis

For Sample 1 data, the Kaiser–Meyer–Olkin measure verified sampling adequacy (KMO = 0.938), and Bartlett’s test of sphericity was significant (χ^2^ = 11506.95, *p* < 0.001). Principal component analysis with oblique rotation yielded four factors with eigenvalues > 1, collectively explaining 62.5% of the total variance. All 24 items loaded satisfactorily on their respective factors (loadings > 0.692) (see Table 4).

**TABLE 4:** Exploratory factor analysis results for the Problem-Posing Ability Scale (*n* = 961).

Item	Factor 1	Factor 2	Factor 3	Factor 4
A22	0.788			
A2	0.764			
A10	0.741			
A18	0.741			
A14	0.739			
A6	0.737			
A23		0.777		
A3		0.736		
A19		0.752		
A15		0.732		
A7		0.716		
A11		0.713		
A4			0.769	
A24			0.770	
A16			0.754	
A20			0.745	
A8			0.715	
A12			0.708	
A21				0.781
A13				0.768
A1				0.796
A17				0.734
A9				0.720
A5				0.692
Eigenvalue	8.505	2.555	2.114	1.826
variance explained (%)	35.44	10.65	8.81	7.61
Cumulative variance explained (%)	35.44	46.08	54.89	62.5

The four factors were labeled:

- Factor 1: Questioning Courage and Initiative (items 2, 6, 10, 14, 18,22)
- Factor 2: Ability to Articulate and Organize Problems (items 3, 7, 11, 15, 19, 23)
- Factor 3: Depth and Criticality of Thinking (items 4, 8, 12, 16, 20, 24)
- Factor 4: Problem Awareness and Sensitivity (items 1, 5, 9, 13, 17, 21)

### 3.3 Confirmatory factor analysis

CFA performed on Sample 2 data supported the four-factor model, demonstrating excellent fit to the data (χ^2^/df = 1.252, CFI = 0.992, TLI = 0.991, RMSEA = 0.021, SRMR = 0.043). Standardized factor loadings ranged from 0.692 to 0.815 (all *p* < 0.001). The four-factor model fit the data significantly better than a single-factor model (see Table 5). Inter-factor correlations ranged from 0.549 to 0.767 (all *p* < 0.001) (see Table 6).

**TABLE 5:** Confirmatory factor analysis fit indices for the Problem-Posing Ability Scale (*n* = 577).

Model	χ ^2^/df	CFI	TLI	RMSEA	SRMR
Four-factor model	1.252	0.992	0.991	0.021	0.043
One-factor model	28.140	0.000	0.000	0.217	0.637

**TABLE 6:** Intercorrelations among factors of the Problem-Posing Ability Scale (*n* = 577).

Factor	1	2	3	4
1. Questioning courage and initiative	0.767	0.734	0.763	0.755
2. Ability to articulate and organize problems	0.672***			
3. Depth and criticality of thinking	0.549***	0.598***		
4. Problem awareness and sensitivity	0.562***	0.599***	0.655***	

***P < 0.001.

### 3.4 Reliability and validity

#### 3.4.1 Reliability

Internal consistency was high for both the total scale and subscales: total Cronbach’s α = 0.94; subscale α ranged from 0.875 to 0.895. Test-retest reliability over a 4-week interval (n = 96) was excellent: total scale *r* = 0.955; subscale *r*-values ranged from 0.914 to 0.924.

#### 3.4.2 Criterion validity

PPAS total and subscale scores correlated significantly with the total and subscale scores of the Williams Creative Tendency Scale (*r* = 0.601–0.903, all *p* < 0.01) (see Table 7).

**TABLE 7:** Criterion-related validity analysis of the Problem-Posing Ability Scale (*n* = 577).

Dimension/scale	Risk-taking	Curiosity	Imagination	Challenge	Creativity total
Questioning courage and initiative	0.601**	0.622**	0.709**	0.613**	0.711**
Ability to articulate and organize problems	0.607**	0.641**	0.740**	0.630**	0.732**
Depth and criticality of thinking	0.615**	0.611**	0.709**	0.635**	0.714**
Problem awareness and sensitivity	0.681**	0.679**	0.740**	0.627**	0.764**
Problem-Posing Ability Scale total	0.774	0.788	0.895	0.774	0.903

**P< 0.01.

## 4 Discussion

### 4.1 Exploring PPAS structure

This study developed and validated a 24-item, four-dimensional Problem-Posing Ability Scale (PPAS) for secondary school students. The scale exhibits strong psychometric properties, including high internal consistency (total Cronbach’s α = 0.94), excellent test-retest reliability (*r* = 0.955), and satisfactory criterion-related validity with the Williams Creative Tendency Scale (*r* = 0.903). Exploratory factor analysis revealed a four-factor structure accounting for 62.5% of the total variance. Confirmatory factor analysis confirmed the model’s robustness, with all fit indices meeting stringent psychometric standards (χ^2^/df = 1.252, CFI = 0.992, TLI = 0.991, RMSEA = 0.021).

The final four-factor structure demonstrates strong theoretical and empirical validity. Theoretically, it integrates cognitive dimensions (e.g., articulation, critical thinking) with non-cognitive elements (e.g., questioning courage and initiative). This aligns with contemporary models of problem-posing ability that emphasize the interplay of affective, motivational, and cognitive components (Zhang et al., 2014). The structure comprising “Questioning Courage and Initiative,” “Ability to Articulate and Organize Problems,” “Depth and Criticality of Thinking,” and “Problem Awareness and Sensitivity” inherently reflects the developmental progression from “daring to ask” to “knowing how to ask” and ultimately to “asking good questions” (Gao et al., 2025). This progression aligns with developmental stages of questioning ability (Hong et al., 2021) and the complete cognitive process from information extraction to problem generation (Christou et al., 2005; Shi, 2025). Moreover, this structure incorporates the essential components of creative thinking, namely fluency, flexibility, and originality (Balka, 1974; Silver and Cai, 1996), which strengthens the conceptual connection between PPA and creative cognition.

### 4.2 Implications

Compared to existing assessment tools, the PPAS offers distinct advantages. Traditional instruments like the Primary Trait Analysis (PTA) often focus primarily on cognitive outcomes such as the quantity, variety, and quality of posed problems (Gao, 2016). In contrast, the PPAS provides a more comprehensive framework by integrating both cognitive and non-cognitive factors. This addresses a significant gap, as motivational factors like questioning confidence are crucial for genuine critical inquiry (Zhang et al., 2014). Furthermore, the PPAS incorporates dimensions relevant to contemporary technological contexts, such as the depth and clarity of thinking required for effective human–AI interaction. This enhances its ecological validity compared to tools developed for specific academic domains (e.g., mathematics or science) (Shi, 2021; Yang et al., 2025). The scale’s item phrasing, refined through student interviews and expert reviews, closely aligns with secondary school students’ authentic language and cognitive contexts, thereby minimizing measurement bias associated with abstract terminology. The PPAS holds substantial potential for diverse practical applications in educational settings.

- *For instructional diagnosis and intervention*: The scale enables educators to identify specific student weaknesses across its four dimensions, facilitating targeted pedagogical strategies. For instance, low scores on “Questioning Courage and Initiative” may indicate a need to foster a safer and more supportive classroom climate (Zhou and Lyu, 2002). Conversely, low scores on “Depth and Criticality of Thinking” can prompt the implementation of critical thinking training (Yang, 2018), while deficiencies in “Ability to Articulate and Organize Problems” might call for focused training in logical expression.
- *For AI-enhanced learning environments*: In the era of generative artificial intelligence, the PPAS can be instrumental in evaluating the quality of student-AI interactions and designing adaptive scaffolds. For example, students who struggle with posing precise queries to AI tools (as reflected in lower scores on articulation or depth) can benefit from guided templates that prompt multi-angle questioning or deeper reflection, thereby enhancing their ability to transform vague needs into high-quality inquiries (Lan and Lin, 2011).
- *For educational policy and research*: At a systemic level, data collected using the PPAS can help identify regional or inter-school disparities in PPA, informing equitable resource allocation and policy decisions (Zhang J. et al., 2025). Furthermore, the scale provides a robust tool for conducting future empirical research, such as investigating the complex mechanisms underlying PPA (e.g., the mediating role of anxiety between shyness and questioning ability) (Zhang et al., 2014), or assessing the efficacy of instructional interventions like project-based learning on specific PPA dimensions (Huang and Sang, 2025).

### 4.3 Limitations and future directions

This study has several limitations that should be acknowledged. First, while the sample was geographically diverse, its distribution across key demographic variables (e.g., school type, region) was not fully representative, which may affect the generalizability of the findings. Second, criterion-related validity was established using a measure of creative tendency. Future studies should incorporate a broader range of external criteria, such as performance on actual problem-posing tasks or teacher ratings. Third, the present version of the PPAS does not incorporate items specifically crafted to assess the capability of posing questions to generative AI tools (e.g., ChatGPT, DeepSeek). This is a skill of increasing importance that should be incorporated in subsequent versions (Hazeyama and Hirai, 2009). Fourth, the cross-sectional design limits causal inference; longitudinal research is needed to test the scale’s sensitivity in tracking developmental changes or measuring intervention effects over time. Finally, conducting cross-cultural comparative studies would be valuable for exploring how cultural contexts influence the manifestation and development of different PPA dimensions (Huang and Sang, 2025).

In conclusion, by constructing a multidimensional model of problem-posing ability and subjecting it to rigorous psychometric evaluation, this study provides a reliable and valid tool for assessing PPA among secondary school students. The PPAS addresses limitations of existing instruments and offers a practical foundation for advancing research and the implementation of a “question-driven” educational paradigm.

## Data Availability

The raw data supporting the conclusions of this article will be made available by the authors, without undue reservation.
